# WINNER: A network biology tool for biomolecular characterization and prioritization

**DOI:** 10.3389/fdata.2022.1016606

**Published:** 2022-11-04

**Authors:** Thanh Nguyen, Zongliang Yue, Radomir Slominski, Robert Welner, Jianyi Zhang, Jake Y. Chen

**Affiliations:** ^1^Informatics Institute in School of Medicine, The University of Alabama at Birmingham, Birmingham, AL, United States; ^2^Department of Biomedical Engineering, The University of Alabama at Birmingham, Birmingham, AL, United States; ^3^Comprehensive Arthritis, Musculoskeletal, Bone and Autoimmunity Center (CAMBAC), School of Medicine, The University of Alabama at Birmingham, Birmingham, AL, United States

**Keywords:** gene prioritization, network expansion, network statistical analysis, pathway analysis, network biology

## Abstract

**Background and contribution:**

In network biology, molecular functions can be characterized by network-based inference, or “guilt-by-associations.” PageRank-like tools have been applied in the study of biomolecular interaction networks to obtain further the relative significance of all molecules in the network. However, there is a great deal of inherent noise in widely accessible data sets for gene-to-gene associations or protein-protein interactions. How to develop robust tests to expand, filter, and rank molecular entities in disease-specific networks remains an ad hoc data analysis process.

**Results:**

We describe a new biomolecular characterization and prioritization tool called Weighted In-Network Node Expansion and Ranking (WINNER). It takes the input of any molecular interaction network data and generates an optionally expanded network with all the nodes ranked according to their relevance to one another in the network. To help users assess the robustness of results, WINNER provides two different types of statistics. The first type is a node-expansion *p*-value, which helps evaluate the statistical significance of adding “non-seed” molecules to the original biomolecular interaction network consisting of “seed” molecules and molecular interactions. The second type is a node-ranking *p*-value, which helps evaluate the relative statistical significance of the contribution of each node to the overall network architecture. We validated the robustness of WINNER in ranking top molecules by spiking noises in several network permutation experiments. We have found that node degree–preservation randomization of the gene network produced normally distributed ranking scores, which outperform those made with other gene network randomization techniques. Furthermore, we validated that a more significant proportion of the WINNER-ranked genes was associated with disease biology than existing methods such as PageRank. We demonstrated the performance of WINNER with a few case studies, including Alzheimer's disease, breast cancer, myocardial infarctions, and Triple negative breast cancer (TNBC). In all these case studies, the expanded and top-ranked genes identified by WINNER reveal disease biology more significantly than those identified by other gene prioritizing software tools, including Ingenuity Pathway Analysis (IPA) and DiAMOND.

**Conclusion:**

WINNER ranking strongly correlates to other ranking methods when the network covers sufficient node and edge information, indicating a high network quality. WINNER users can use this new tool to robustly evaluate a list of candidate genes, proteins, or metabolites produced from high-throughput biology experiments, as long as there is available gene/protein/metabolic network information.

## Introduction

Gene prioritization from large-scale omics projects is a central topic in disease biology (Huang H. et al., 2009). Manual searches of the literature and publicly annotated databases (Gene Ontology et al., 2013; Kanehisa et al., 2017; Tyner et al., 2017) for genes associated with a particular disease or biological process can be biased, because they are limited to existing knowledge. Sifting hundreds and thousands of gene or genetic variations associated with genes from genomic studies can also be daunting (Moreau and Tranchevent, 2012), e.g., even for a user to search for genes associated with cardiac arrhythmia (Rajab et al., 2010) within a 2-Mb region of chromosome 17 may return 77 candidate genes. For many biologists, the lack of ranking of genes based on biological relevance of disease context is an experience analogous to the pre-Google days of Internet search of web content. With influx of data from large-scale sequencing projects (Schlotterer et al., 2014), bioinformatics users increasingly count on good gene prioritization to help them generate biological hypotheses (Chen et al., 2006a; Hale et al., 2012), find potential disease biomarkers (Saha et al., 2008; Zhang and Chen, 2010, 2013), and identify candidate drug targets (Chen et al., 2006b, 2013; Li et al., 2009; Muhammad et al., 2017). However, as datasets continue to become larger and more heterogeneous, statistical (Subramanian et al., 2005; Aerts et al., 2006; Cantor et al., 2010) and text-mining (Krallinger et al., 2008; Liu et al., 2015; ElShal et al., 2016) approaches to gene prioritization lack sufficient precision in the biological knowledge context. For example, surveys of PAGER (Yue et al., 2018) for genes associated with the response of breast cancer to doxorubicin treatment may retrieve more than 2,000 statistically significant genes with MSigDB (Liberzon et al., 2015), or 234 candidate genes with the online text-mining platform Beegle (ElShal et al., 2016). The use of statistical *p*-values to prioritize retrieved genes can mislead biology users who assume statistical significance in samples equate the gene's true biological significance against one another in the experiment (Kim and Bang, 2016).

To overcome the limitations gene prioritization in practice, bioinformatics researchers have developed gene network models with which they perform knowledge-based gene prioritization and novel candidate genes identification (Chen et al., 2006a; Cowen et al., 2017). A molecular network consists of nodes (e.g., proteins) linked by edges that represent the pairwise interactions between nodes, forming a convenient computational model that is easy to interpret and has been widely used to discover (and rediscover) disease-specific genes and potential targets for treatment (Chen et al., 2009; Wu et al., 2009; Erten et al., 2011; Gottlieb et al., 2011; Guney and Oliva, 2012; Singh-Blom et al., 2013; Smedley et al., 2014; Peters et al., 2017; do Valle et al., 2018). Network-based methods also enable researchers to integrate data from a wide variety of sources, including analyses of gene-gene similarity (Alvarez-Ponce et al., 2013), proteomic interactions (Rolland et al., 2014), and regulatory pathways (Li and Campos, 2015); however, the results of prioritization strongly depend on the input gene list (Antanaviciute et al., 2015), and the list is often derived from existing databases that may lack important genes because of statistical errors or human errors during annotation. For example, acetylcholinesterase (ACHE), which is commonly associated with β-amyloid plaques and neurofibrillary tangles in the brains of patients with Alzheimer's Disease (AD; Talesa, 2001), is not among the annotated genes for AD in the KEGG database (Kanehisa et al., 2017). Input lists may also be compromised by redundancy, which can be generated from at least two sources: (1) the inclusion of genes that were falsely identified during the statistical analysis of an experiment (Yu et al., 2017), and (2) when, in an attempt to increase comprehensiveness, the list is expanded to include the gene for a “hub” protein that interacts with dozens, or even hundreds, of other proteins [e.g., ubiquitin C binds to 4,658 other molecules (Chen et al., 2017)] and, consequently is unlikely to be specific for the phenotype of interest. Furthermore, the statistical significance of a ranking is typically calculated *via* comparison to the rankings from a randomized version of the original network, but since the randomized network is often created by adding or deleting a small number of gene-gene interactions (i.e., increasing noise), or *via* total network permutation (Xie et al., 2015; Guala and Sonnhammer, 2017), much of the topology of the original network may be lost.

### Related works

According to Bromberg (2013), molecular-interaction-based disease gene prioritization started in the early 2000's by pioneering techniques such as G2D (Perez-Iratxeta et al., 2002). In principle, statistical analysis of the patients' genetic data yields 100's of disease-associated genes. These genes often belong to an interaction network (Sun and Zhao, 2010), which is also called a “disease pathway.” Assume that the disease phenotypes occur due to a disturbance at any point of the pathway, then disturbing the “most influential” genes is the most likely reason leading to the disease. Then, having a good disease pathway, network ranking algorithms, especially the eigenvector-based [Random Walk (Smedley et al., 2014) and PageRank (Page et al., 1999)] and centric-based [betweenness centrality (Newman, 2005)] can be used to prioritize the genes. Also, this idea can be applied to analyze key regulators in non-disease-specific biological processes. However, the pathways are usually incompleted: new disease regulators are still not discovered or some interaction among disease-associated genes are not yet shown (Bromberg, 2013). Therefore, the ranking techniques are required to extend the interaction network beyond the known disease-associated genes. Recent gene prioritization techniques have this ability. For example, DIAMOnD (Ghiassian et al., 2015) built a large network comprising genes related to 70 diseases, clustered the large network into multiple network modules, then assigned the network module to a disease; here, in the same module, genes not related to the disease module are added (extended) into the disease-specific network-module for prioritization. Ingenuity Pathway Analysis (Kramer et al., 2014) extended the disease-specific pathway by statistically estimating the likelihood of how a new gene interacts with the known disease-related gene. In Node2Vec (Grover and Leskovec, 2016; Peng et al., 2019), a “global gene network,” which includes the known disease-specific genes, their direct interacting genes, and indirect interacting ones (optionally) was constructed; then, each gene is represented by a numerical vector having a fixed-length dimension to allow computing the cosine similarity between a known disease-specific gene and another gene; so, the extension can be made by choosing the genes having high cosine similarity to any of the disease-specific ones. Or, in GenePANDA (Yin et al., 2017), given a “global gene network” (similar to Node2Vec), for a specific gene, the average distance between itself and any other gene in the “global” network was subtracted by the average distance between itself and the known disease-specific genes; then, this difference was used to rank the genes.

Besides the network-based approach, gene prioritization could be performed using text mining and similarity profiling approaches (Yin et al., 2017). In the text mining approach, it is hypothesized that important genes are more likely to be mentioned in an article than non-important ones. Therefore, text mining tools, such as aBandApart (Van Vooren et al., 2007) and Gene Prospector (Yu et al., 2008), emphasize efficient queries in MEDLINE and other large literature collections to find important disease-specific genes. However, these approaches may not find important genes when the disease is not yet well-researched or when a new disease model (i.e., a new cell line or new organoid) is built to represent the disease. On the other hand, similarity profiling defines the similarity among the genes according to the disease-related information; then, if a novel gene shares a high similarity with genes that are known to be important, the novel gene will be ranked highly. For example, Endeavor (Aerts et al., 2006) and ToppGene (Chen et al., 2009) integrated multiple disease-omic databases by a machine-learning model; the model was trained to classify between the known-important genes and non-important genes; the model will produce a ranking score reflecting how important a novel gene is, respecting the already known ones. Meanwhile, the disease-specific gene expression and correlation matrix can be clustered or latent-based represented, such as in Pinta (Nitsch et al., 2011), Maxlink (Guala et al., 2014), and Genefriends (van Dam et al., 2012), where the well-known disease-specific genes are expected to concentrate in one or a few clusters/latent modules, and the novel genes in these clusters or modules would be ranked highly.

Here, we introduce a new ranking method, Weighted In-Network Node Expansion and Ranking (WINNER), that addresses many of the current limitations of network-based gene prioritization methods. As with PageRank (Winter et al., 2012) and many other gene prioritization techniques, the ranking engine of WINNER uses random-walk principles (Zhao et al., 2015). However, WINNER was designed to address the following three specific network biology tasks: (1) perform gene prioritization in a weighted biomolecular association network, (2) identify upstream regulators and targeted genes (i.e., “upstream” ranking), or (3) identifying downstream effector molecules that are specific for a particular disease or phenotype (“downstream” ranking). WINNER can generate a ranking score for each input gene, derive optional genes that are “expanded” from the original seed gene lists, and provide two different statistic for users (1) the gene expansion *p*-value (*p*_*e*_) for adding a gene to the network, which addresses both incomprehensiveness and redundancy; and (2) the gene ranking *p*-value (*p*_*r*_), which represents the significance of the ranking when compared to the randomized network. Furthermore, we found that compared to total network permutation (Xie et al., 2015; Guala and Sonnhammer, 2017), preserving the modularity randomization (Cowen et al., 2017) produces a randomized network that is topologically similar to the original network and yields a more normal distribution of ranks (Espinoza, 2012). We further demonstrated the benefit of WINNER in omics study result interpretations with the following case studies: (1) ranking genes that are genetically associated with Alzheimer's disease (AD); (2) ranking breast-cancer survival-related genes (Lanczky et al., 2016); (3) ranking differentially expressed genes involved in myocardial injury in pigs for their potential roles in myocardial regeneration (Eschenhagen et al., 2017). In all these studies, we discuss how our prioritization score and statistic associated with high-ranked genes enable biology users to derive new insights and hypotheses worth further experimental investigations.

## Methods

For this work, we postulated (1) that the seeded (i.e., input) genes consist of (but are not limited to) differentially expressed genes identified in a wet-lab experiment, genes in a well-curated pathway, and phenotype-associated genes mined from the literature; and (2) that genes added to the expanded network (i.e., “expansion genes”) would have significantly more interactions with seeded genes (i.e., “seeded interactions”) than with non-seeded genes. WINNER begins with the set of seeded genes and a collection of gene-gene interactions, iteratively applies network ranking for gene prioritization, and expands the ranked list of genes one gene at a time (Supplementary Figure 1). Each gene-gene interaction has a confidence score (scaled between 0 and 1), which is commonly included in interactome databases (Chatr-Aryamontri et al., 2013; Szklarczyk et al., 2015); however, if a confidence score is not available, then the confidence score is set to 1 for all interactions. Network ranking is first applied to the seeded genes and the interactions among them (*S*_0_ metric, Equation 1); then, genes adjacent to the seeded genes are filtered for significant interactions with the seeded genes (*p*_*e*_) to identify candidates for the expanded network. The identified candidate is added to the ranked list, and network ranking is re-applied to initiate the next iteration of the cycle. A more detailed description of each step is provided below.

### Ranking genes in the network by WINNER

#### Undirected networks

Given a gene-gene association network, the genes are ranked as in Supplementary Video 1. First, WINNER assigns an initial score (*S*_0_) to the genes, according to Yue et al. (2017):


(1) $$\begin{array}{l} S_{0}\left(i\right)=e^{2\ln\left(w\left(i\right)\right)-ln\left(I\left(i\right)\right)} \end{array}$$

where *i* represents the gene index, *w*(*i*) is the sum of the confidence scores (normalized to between 0 and 1) for all gene-gene interactions associated with *i*, and *I*(*i*) is the number of gene-gene interactions associated with *i*. Here, larger confidence scores imply stronger associations. Second, WINNER iteratively updates the gene score by applying the Random Walk technique (Page et al., 1999):


(2) $$\begin{array}{l} S_{t}\left(i\right)=\left(1-\sigma \right)\times S_{0}\left(i\right)+\sigma \times \underset{\forall j}{\sum }\frac{c\left(j,i\right){\times S}_{t-1}\left(j\right)}{w\left(j\right)} \end{array}$$

where *s* is the random walk damping parameter [set to *s* = 0.85 as described (Page et al., 1999)], *c*(*j, i*) represents the confidence score of the interaction between gene *i* and gene *j*, and *t* is the index of iteration (starting at 1); *S* = 0 for genes that are outside the network but appear in the collection of gene-gene interactions. PageRank theory (Page et al., 1999) demonstrates that *S*_*t*_ converges (|*S*_*t*_ − *S*__*t*−_1_|^®^0) if *t* is large enough, so the iterative cycle was continued until |*S*_*t*_ − *S*__*t*−_1_| < 0.001.

#### Directed networks

Directed networks, such as networks of regulatory pathways, include more annotation than undirected networks. Thus, we adapted the definitions of terms in Equations 1, 2 so that WINNER could be used to (for example) infer upstream regulatory and downstream effector genes (Kramer et al., 2014). For “upstream” ranking, *i* is the regulatory gene and *j* is the gene regulated by *i*; thus, *w*(*i*) is the sum of the confidence scores for all gene-gene relationships that *i* regulates, *I*(*i*) is the number of gene-gene relationships regulated by *i*, and *c*(*j, i*) is the confidence score for the regulation of *j* by *i*. For “downstream” ranking, *i* is the regulated gene and *j* is the gene that regulates *i*; thus, *w*(*i*) is the sum of the confidence scores for all gene-gene relationships in which *i* is regulated, *I*(*i*) is the number of gene-gene relationships in which *i* is regulated, and *c*(*j, i*) is the confidence score for the regulation of *i* by *j*.

#### Statistical significance of gene ranking

To evaluate the statistical significance (*p*-value) of the gene ranking, we determined how likely the converging result of *S* (by default, *S*_200_) in Equations 1, 2 is higher than in random networks. Randomization was performed in Matlab with degree-preservation (Espinoza, 2012; Tiong and Yeang, 2019) to maintain the topological characteristics of the original gene-gene network; however, the technique only generates unweighted relationships, so weights were randomly assigned from the distribution of relationship weights in the original network. One thousand random networks were generated, and the ranking scores (*S*_200_) of the genes in the random networks were normally distributed (as validated *via* the Chi-square goodness-of-fit test). Thus, the ranking *p*-value (*p*_*r*_) for each gene *i* was calculated by using the normal distribution [*m*(*i*), *s*(*i*)] parameter estimation (Bowman and Azzalini, 1997):


(3) $$\begin{array}{l} p_{r}\left(i\right)=\{\begin{array}{c} \int_{-\infty }^{S_{200}\left(i\right)}\frac{1}{\sigma \left(i\right)\sqrt{2\pi }}e^{-\frac{{\left(x-\mu \left(i\right)\right)}^{2}}{2\sigma^{2}}}dx\;if\;S_{200}\left(i\right)<\;\mu \left(i\right)\\ \int_{S_{200}\left(i\right)}^{\infty }\frac{1}{\sigma \left(i\right)\sqrt{2\pi }}e^{-\frac{{\left(x-\mu \left(i\right)\right)}^{2}}{2\sigma^{2}}}dx\;if\;S_{200}\left(i\right)>\;\mu \left(i\right) \end{array} \end{array}$$

which is equivalent to computing the two-tailed *p*-value for a normal distribution.

### Filtering candidates for expansion

We chose two hypergeometric tests that are common practice in annotation (Huang et al., 2009). First, we tested the likelihood of the candidate expansion gene having a seeded interaction relative to its total number of interactions. Second, we tested the likelihood of the candidate expansion gene having seeded interactions relative to the seeded interactions of its most similar seeded gene, with similarity determined by node degree. Thus, we calculated two *p*-values for each expansion gene *j* from the “overrepresented” point of view (Beissbarth and Speed, 2004; terms are defined in Supplementary Figure 2):

Test 1:


(4) $$\begin{array}{l} p_{1e}\left(j\right)=\overset{\min\left(n,K\right)}{\underset{l=k\left(j\right)}{\sum }}\frac{\left(\begin{array}{c} K\\ l \end{array}\right)\left(\begin{array}{c} N-K\\ n-l \end{array}\right)}{\left(\begin{array}{c} N\\ K \end{array}\right)} \end{array}$$

Test 2:


(5) $$\{\begin{array}{c} p_{2e}\left(j\right)=\overset{\min\left(n,K\right)}{\underset{l=k\left(j\right)}{\sum }}\frac{\left(\begin{array}{c} K\\ l \end{array}\right)\left(\begin{array}{c} N-K\\ n-l \end{array}\right)}{\left(\begin{array}{c} N\\ K \end{array}\right)}\;if\;N>K\\ p_{2e}(j)=1-\overset{\min\left(n,K\right)}{\underset{l=0}{\sum }}\frac{\left(\begin{array}{c} N\\ l \end{array}\right)\left(\begin{array}{c} K-N\\ k-l \end{array}\right)}{\left(\begin{array}{c} K\\ N \end{array}\right)}\;if\;N<K \end{array}$$

in which the double-line bracket operator represents the combination operator:


(6) $$\begin{array}{l} \left(\begin{array}{c} N\\ K \end{array}\right)=\frac{N\left(N-1\right)\left(N-2\right)\ldots \left(N-K+1\right)}{K\left(K-1\right)\left(K-2\right)\ldots 1} \end{array}$$

Genes for which both *p*_1e_(*j*) < 0.05 and *p*_2e_(*j*) < 0.05 were chosen as candidates for expansion. Thus, the expansion *p*-value (*p*_*e*_) for each gene *j* is defined by the equation *p*_*e*_(*j*) = max [*p*_1e_(*j*), *p*_2e_(*j*)].

### Selecting one candidate for expanded ranking

Since there will likely be more than one candidate expansion gene remaining after filtration, WINNER estimates which of the candidates should be added to the network by calculating an expansion score (*e*) from the confidence score of the interaction between the candidate gene and the ranked genes, and the ranking score (*S*) of the ranked genes:


(7) $$\begin{array}{l} e\left(i\right)=\sum \frac{c\left(i,j\right)S\left(j\right)}{W\left(j\right)} \end{array}$$

Where *i* is the candidate expansion gene, *j* represents all seeded genes that interact with the candidate expansion gene, and *W*(*j*) is the sum of the confidence scores for all interactions involving all seeded genes. Note that *W*(*j*) differs from *w*(*j*) in Equation 2, because *w*(*j*) is restricted to interactions among ranked genes.

### Informatics databases and benchmarking metrics

Correlations among WINNER, PageRank (Winter et al., 2012), dual node-edge ranking (Wang et al., 2015), eigenvector centrality, betweenness centrality, node degree, and clustering coefficient (Newman, 2008) were evaluated by computing the linear correlation coefficients and *p*-values with Matlab (Neupane and Kiser, 2018).

For analyses of upstream and downstream genes (directed network), genes were distributed into layers *via* the breadth-first-search approach, and groups of genes that formed a self-contained cycle were treated as a single node. Results were visualized with boxplots. In each pathway, the gene rank numbers were converted into percentile format: the first rank (number 1) was converted to 100% percentile, while the last rank was converted to 0% percentile. The percentile format allowed boxplot aggregation from multiple pathways, where the different pathways had different number of genes.

Experiments demonstrating the general topological and biological significance of the WINNER ranking were conducted with the small gene set associated with AD from KEGG release 50 (2009) (Kanehisa et al., 2010) and with undirected gene-gene interactions from HAPPI version 1.0 (Chen J. Y. et al., 2009). Rankings of upstream regulators and downstream effectors were conducted with all cancer disease pathways in KEGG release 85 (Kanehisa et al., 2017; Tessier et al., 2018) and gene-gene regulatory relationships from STRING v.10.5 (Szklarczyk et al., 2017).

The effectiveness of WINNER for identifying network-expansion genes was evaluated by using KEGG release 50 [stored in PAGER 1.0 (Yue et al., 2015)] as the input with interactions of all types (without directionality) from HAPPI v.2.0 whose confidence scores exceeded 0.75 (Chen et al., 2017), and then determining how closely the expanded network matched the updated KEGG release 85 (Kanehisa et al., 2017). An analogous experiment was conducted with Ingenuity Pathway Analysis (IPA), which (in theory) can be used for both upstream and downstream expansion and HAPPI v.2.0 (Kramer et al., 2014) for comparison. Precision, recall, and F1 scores were calculated *via* the following equations:


(8) $$\begin{array}{l} precision=\frac{\left|E\cap U\right|}{\left|E\right|} \end{array}$$

(9) $$\begin{array}{l} recall=\frac{\left|E\cap U\right|}{\left|E\right|} \end{array}$$

(10) $$\begin{array}{l} F1=\frac{2\times precision\times recall}{precision+recall} \end{array}$$

where E is the set of expansion genes determined by Winner or IPA and U is the set of genes present in KEGG release 85 but not in KEGG release 50.

The biological relevance of our rankings was evaluated by (1) determining whether the top-ranked genes from WINNER ranking of the KEGG breast cancer pathway (Kanehisa et al., 2017; https://www.genome.jp/kegg-bin/show_pathway?hsa05224) were included among the genes correlated with survival in 3951 Breast Cancer patients (Gyorffy et al., 2010); and (2) by ranking the set of differentially expressed genes from a study of myocardial regeneration in neonatal pigs (Zhu et al., 2018) with WINNER and determining whether the top-ranked genes could contribute to cardiac repair and cardiomyocyte proliferation. For the analysis of breast-cancer survival genes, we calculated the ratio of the number of genes that were both significant (survival *p*-value < 0.05) in the breast cancer study (Gyorffy et al., 2010) and highly ranked by WINNER (i.e., scored above a defined threshold) to the number of highly-ranked genes.

### Network randomization and testing for ranking normal distribution in random networks

In WINNER, given a network (also called the original network), we examined the following network randomization approaches to evaluate which network randomization approach was the most suitable for computing the ranking *p*-value for each gene:

- Total rewiring (also called total network permutation; Waksman, 1968). To implement this approach, for each interaction (edge) in the original network, we randomly changed the two genes (node) connecting through this edge. Therefore, this approach preserves the number of interactions, yet it totally changes the network and gene topology.
- Randomly drawing a new network such that each gene's degree is the same to what it is in the original network (also called preserving degree; Rao et al., 1996). A gene degree, in simple description, is the number of other genes connecting to the gene in the network.
- Randomly drawing a new network with the same modularity to the original network (also called preserving modularity). We implemented this strategy according to the network modularity definition in Newman (2006). Modularity measures likely the network can be partitioned into clusters of interacting genes.
- Randomly adding 5% new interactions into the original network. These interactions were not reported in the gene-gene interaction databases.
- Randomly removing 5% of the interactions from the original network.

For each network randomization approach, starting from the same original network, we repeated 10,000 times, yielding 10,000 different random networks. Then, applying WINNER (and other ranking algorithms) yielded 10,000 random ranking results for each gene. We tested whether these random rankings followed a normal distribution using chi-square goodness of fit test (chi2gof)^1^ in Matlab. In this test, the smaller chi-square (chi2) indicates that the rankings are more naturally distributed.

### Literature validation using co-citations from PubMed

Important disease-specific genes are often co-mentioned in a research article. Therefore, to demonstrate the significance of the genes related to a disease, we applied a co-citations from the NCBI e-utils application programming interface (API; Sayers, 2008) that implements semantic searches of PubMed abstracts to report biomedical literature citations (https://eutils.ncbi.nlm.nih.gov/entrez/eutils/esearch.fcgi?). We applied “pubmed” as input of database and the concatenated string of the candidate gene and the disease name as input of terms. To identify the co-citation support for the winner scores, we separated the genes into two categories, with literature co-citation (*k* = 0) or without literature co-citation (*k* > 0) to find the differences between the winner scores. We applied the Kruskal-Wallis test to report *p*-values.

### Biomedical case studies, data, and preprocessing

#### Cardiac regeneration dataset

For the cardiac regeneration case study, the bulk-RNA expression dataset was obtained from Zhang et al. (2020). Briefly, two groups of pig hearts were sent for sequencing when they reached postnatal days (P) 7, 14, and 28. In the first group, the pigs underwent myocardial infarction (a heart attack model) on the postnatal day 1, then their heart fully recovered to normal cardiac functionality with no scar. In the second group, the pig did not undergo injury (sham control). For each group at each day (P7, P14, or P28), three pigs were sequenced. The bulk-RNA data were processed by applying trim-galore (Krueger, 2015) for trimming the fastQ read, then STAR package v2.5.2 for mapping to Pig genome (Dobin et al., 2013), then the RNA transcripts were counted using HtSeq version 0.6.1 (Anders et al., 2015). The gene expression was normalized, and fold-change was calculated using Deseq2 software (Love et al., 2014). Due to the small sample size (*n* = 3), the *p*-values for differentially expressed genes, compared between two groups at P7, P14, and P21, were calculated using the approach in Bian et al. (2021). After calculating and comparing two groups at these three different postnatal time points, this process yielded 276 seed genes as input for WINNER. Then, these genes were queried in HAPPI v2 database (Chen et al., 2017) to build their interacting network. These gene lists, their interaction, and WINNER results were summarized in Supplementary Tables 1, 2.

#### Data processing of triple negative breast cancer (TNBC)

Triple negative breast cancer (TNBC) has been found in 15% of breast cancer cases and is characterized by the tumor cells lacking the expression of the following: epidermal growth factor 2 (HER2), estrogen receptor (ER), and progesterone receptor (PR; Liu et al., 2014; Ueda et al., 2019). Unfortunately, because of its nature, TNBC has a poorer prognosis than other types of breast cancers and treatment options are limited (Xia et al., 2014; Eltohamy et al., 2018; Lu et al., 2020). While TNBC markers are already well-studied, finding the key disease regulators and promising targeted genes is still challenging (Nedeljkovic and Damjanovic, 2019). Therefore, we applied WINNER to explore novel answers for this question.

We took the triple negative breast cancer candidate genes from the University of Alabama at Birmingham Cancer data analysis Portal (UALCAN) database (Chandrashekar et al., 2022). In the comparison between the 116 triple negative breast cancer samples and 114 normal samples, UALCAN provided the top 250 up-regulated genes and 250 down-regulated genes selected by the *t*-test *p*-value. Next, we retrieved the Protein-Protein Interaction (PPI) using the medium confidence (score ≥ 0.4) and extended 100 genes using the STRING database. We performed WINNER and generated the gene ranking and *p*-values (Supplementary Tables 3, 4).

#### PubMed co-citation analysis of the WINNER ranked genes

We hypothesize that important disease-specific genes are often co-mentioned in a research article (Olsen et al., 2014); if so, WINNER high-ranking genes tend to be more co-cited in the literature than the low-ranking ones. Therefore, to demonstrate the significance of the genes related to a disease, we applied co-citations from the NCBI e-utils application programming interface (API; Sayers, 2008) that implements semantic searches of PubMed abstracts to report biomedical literature citations (https://eutils.ncbi.nlm.nih.gov/entrez/eutils/esearch.fcgi?). We applied “pubmed” as an input of the database and the concatenated string of the candidate gene and the disease name as input of terms. To identify the co-citation support for the winner scores, we separated the genes into two categories, the WINNER significant ranked genes (*p*-value ≤ 0.05) or WINNER non-significant ranked genes (*p*-value > 0.05) to find the differences between the co-citations. We applied the Kruskal-Wallis test to report *p*-values to test differences of co-citations between significant and non-significant genes.

#### Pathway level assignment

We retrieved significantly enriched pathways from PAGER 2.0 database (Yue et al., 2018) using WINNER highly ranked genes with *p*-values ≤ 0.05. We applied the parameter set as follows. The data sources were KEGG, WikiPathway, BioCarta, NCI-Nature Curated, Reactome, Protein Lounge, and Spike, the similarity was set to be 0.05, and FDR was set to be 0.01. We constructed the regulatory (r-type) PAG-to-PAG network using the default r-type relationship score cutoff (=1). We performed a 5-step procedure in the pathway level assignment. Firstly, we calculated shortest paths among the pairwise r-type PAG-PAG relationships. Secondly, we extracted the longest shortest path and assigned levels of pathway from the upstream to the downstream pathway using 1 to *n*. Thirdly, we expanded the level assignment to the using shortest distances, such as the current pathway is level m, the shortest distance between the expanded pathway in the upstream to the current pathway is 2, the expanded pathway level will be assigned by m-2. Fourthly, we took the average of the levels assigned to pathways. Fifthly, we repeated the steps three and four until all the pathways had been assigned.

#### The correlation analysis of WINNER ranking and the enriched pathways using the exponential scale of top gene bins

Firstly, we segregated the WINNER significant genes into 2^*x*^ bins. Secondly, we took the top 2^*x*^ bins (x is [1, X]) and merge the genes to perform the enrichment analysis. Thirdly, we had the pathways enriched in the top 2^*x*^ gene bins minus the pathways enriched in 2^1^, …,2^*x*−1^ to seek the add-on pathways enriched in the top 2^*x*^ gene bins. Fourthly, we mapped the levels from the r-type pathway-to-pathway relationships to the add-on enriched pathways in each top 2^*x*^ gene bins, and plotted the curve of pathway levels vs. the gene bins. Meanwhile, we performed the Pearson correlation analysis to report the correlation coefficient between the pathways' levels and gene bins.

## Results

### Characteristics of WINNER ranking

#### WINNER ranking of undirected networks

**Figure 1:**
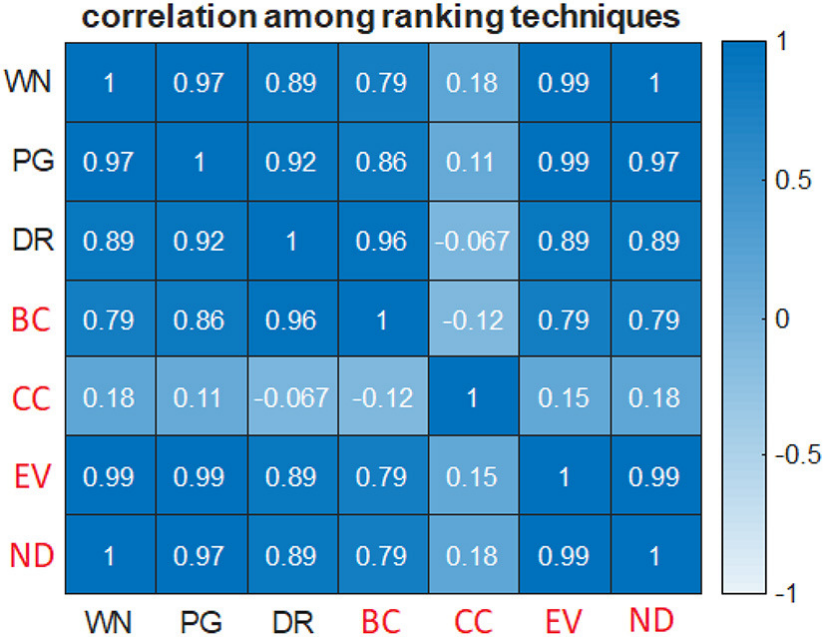
WINNER gene prioritization is well-correlated with other ranking techniques and most network topological metrics. Genes in the KEGG AD pathway were ranked *via* WINNER (WN), PageRank (PG), Dual Node-edge Rank (DR), Betweenness Centrality (BC), clustering coefficient (CC), eigenvector centrality (EV), and node degree (ND); then, the correlation coefficients for all pairwise comparisons between ranking methods were calculated *via* Pearson's correlation.

When genes in the KEGG [release 50, stored in the PAGER 1.0 database (Yue et al., 2015)] AD pathway (Supplementary Figure 3) were ranked *via* WINNER gene prioritization, our results were strongly correlated with those obtained *via* analyses of both eigenvector (Newman, 2008; *p* = 1.45 × 10^−39^) and node-betweenness (Newman, 2008; *p* = 1.67 × 10^−11^) centrality, but not with the clustering coefficient (Newman, 2008; *p* = 0.22). Similar patterns of correlation were obtained with two other state-of-the-art network-based ranking techniques, PageRank (Winter et al., 2012), eigenvector (Newman, 2008), betweenness centrality (Newman, 2005), and dual node-edge ranking (dual rank; Wang et al., 2015) (Figure 1), and all three ranking techniques were strongly correlated with node degree. Notably, the clustering coefficient, but no other metric or technique, failed to identify some of the most important markers for Alzheimer's, including Amyloid Beta Precursor Protein (A4 or APP; Jonsson et al., 2012), Caspase 8 (CASP8; Wei et al., 2002), Caspase 3 (CASP3; D'Amelio et al., 2011), and Presenilin 1 (PSN1; La Bella et al., 2004). Thus, WINNER was at least equivalent to other network topological metrics and well-established prioritization techniques for ranking genes in undirected biological networks.

**Figure 2:**
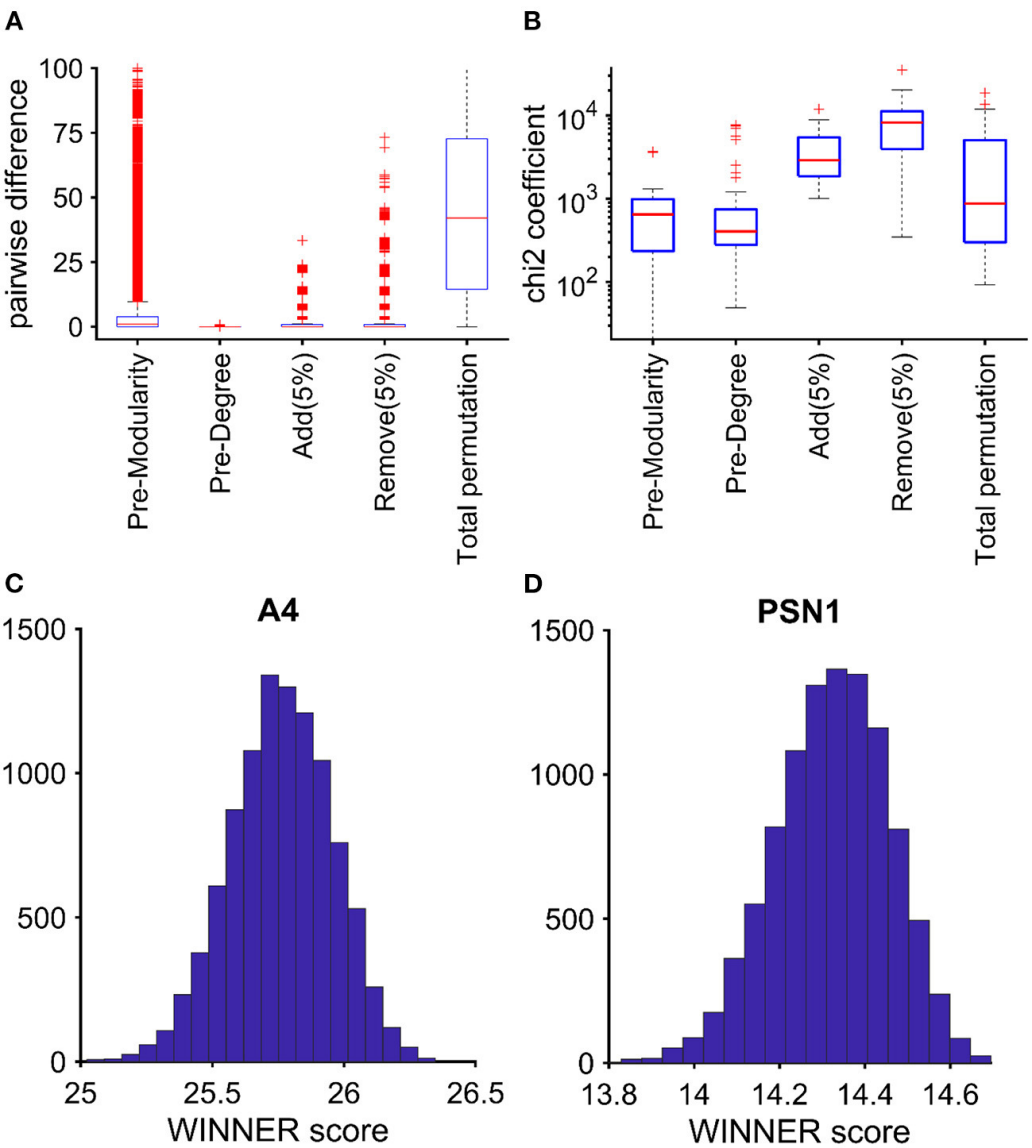
With WINNER, Node-degree–preservation and modularity preservation yields more normally distributed randomized networks. Genes in the KEGG AD pathway were ranked *via* WINNER; then, the ranked networks were randomized *via*: preserving node degree (Pre-Degree), preserving modularity (Pre-Modularity), adding 5% interactions [Add (5%)], removing 5% of the interactions [Remove (5%)], and total network permutation. **(A)** The (pairwise) difference between the original network ranking score and the random network ranking score; smaller difference implies the random network approach is more likely to preserve the original network topology. **(B)** Chi-square (chi2coef) coefficient in chi2gof test (https://www.mathworks.com/help/stats/chi2gof.html). Smaller chi2coef implies that the random ranking is more normally distributed. The (+) signs in the boxplots imply outliners (outside 2 and 98% percentiles). Under random network by preserving node degree, WINNER ranking distributions are in bell-shape for two important AD-related genes: A4 **(C)** and PSN1 **(D)**.

**Figure 3:**
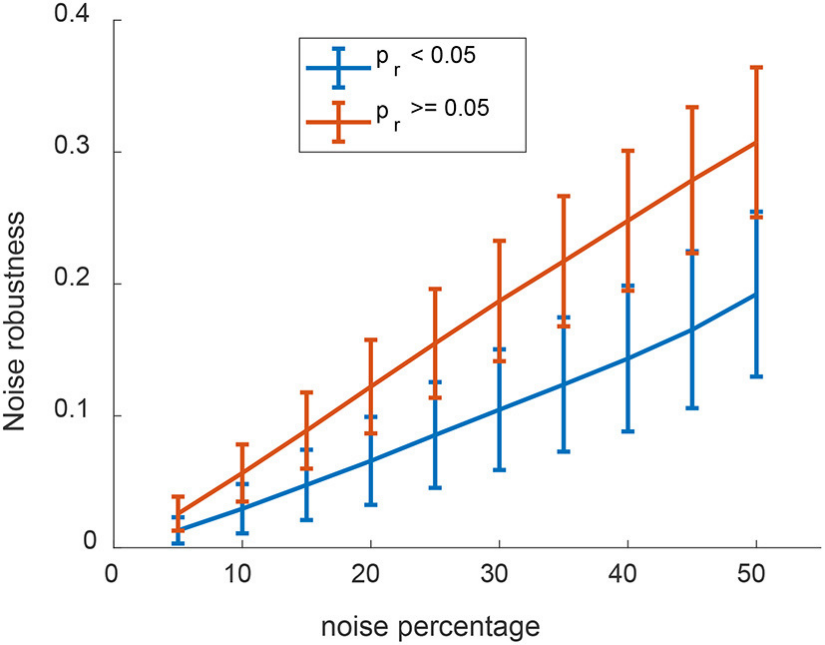
The WINNER ranking *p*-value (*p*_*r*_) is robust to the addition of noise (STATS?). Genes in all KEGG pathways were ranked *via* WINNER, and WINNER ranking *p*-values (*p*_*r*_) were calculated, after varying degrees of noise were added to the network; then, noise robustness was compared for genes with *p*_*r*_ < 0.05 and *p*_*r*_ ≥ 0.05 by determining the likelihood that the gene's ranking changed by 10 or more upon the addition of noise.

The strong correlation between the WINNER and node-degree rankings prompted us to preserve the node degree and modularity during randomization. Examining the AD-associated genes network, the pairwise rank differences between the original network and the total-permutation random network were significantly large (Figure 2A). When the difference between the random ranking and the original ranking is too large, the random network topology would be too different from the original network topology; thus, the random ranking may not be suitable to test statistical significance of the original ranking. Besides, when compared to other randomization techniques (total network permutation, preserving modularity, or adding/removing 5% of edges), the distribution of rankings of AD-associated genes in the degree-preserved randomized network was significantly more normally-distributed (Figure 2B). Furthermore, when examining the ranking distributions of two important AD-associated genes A4 and Presenilin 1 (PSN1; Figures 2C,D), it was clear that their distributions had the bell-shape. Thus, rather than relying on the empirical *p*-value (Cornish et al., 2018) for gene rankings, we generated 1,000 node-preserved randomized networks and calculated a ranking *p*-value (*p*_*r*_) for all genes in all KEGG pathways. Notably, the rankings were much less likely to change in response to the addition of noise for genes with *p*_*r*_ < 0.05 than for genes with *p*_*r*_ ≥ 0.05, especially as the amount of noise increased (Figure 3). These observations suggest that when randomized networks are generated with node-degree preservation, fewer randomizations may be required to achieve adequate precision, and fewer noise simulation may be necessary to evaluate the robustness of the rankings.

**Figure 4:**
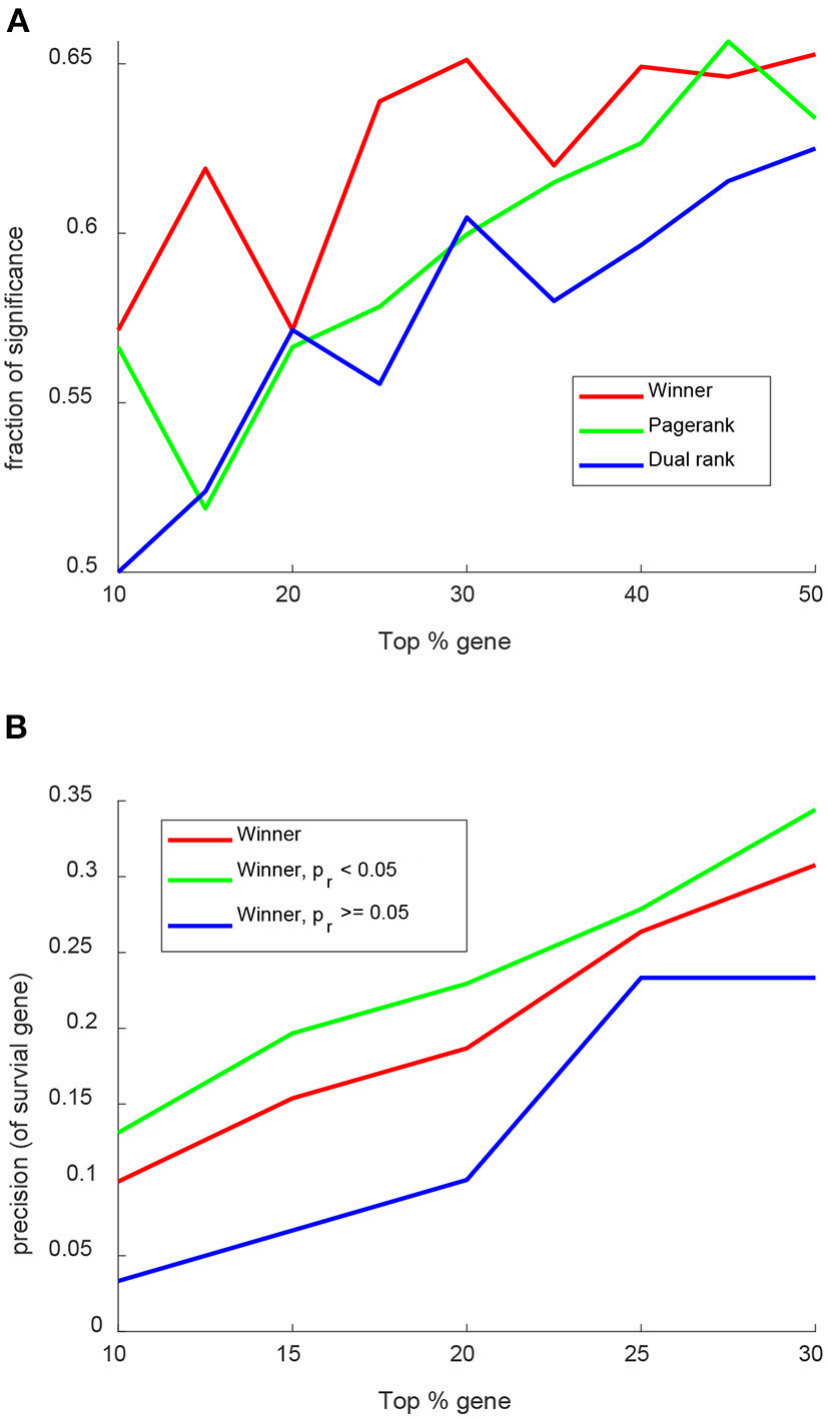
WINNER gene prioritization more accurately identifies the relationship between breast-cancer genes and patient survival. Genes in the KEGG breast-cancer pathway were ranked *via* WINNER, PageRank, and Dual Rank, and the significance of each gene's relationship to patient survival was determined with an online Kaplan-Meier plotting tool. **(A)** The proportion of genes that were significantly (*p* < 0.05) related to breast-cancer survival was determined for the top 0-50% of ranked genes. **(B)** The precision of the WINNER ranking of genes for breast-cancer survival (Bland and Altman, 1998) was compared for the top 0–30% of ranked genes with *p*_*r*_ < 0.05 and *p*_*r*_ ≥ 0.05.

The accuracy of WINNER gene prioritization was evaluated by ranking genes in the KEGG breast cancer pathway (https://www.genome.jp/kegg-bin/show_pathway?hsa05224) and then determining whether the top-ranked genes correlated with the genes' effect on survival for patients with breast cancer, as estimated with an online Kaplan-Meier (Bland and Altman, 1998) tool that calculates the breast-cancer survival rates associated with more than 6,000 genes (Gyorffy et al., 2010). The KEGG breast cancer pathway contains 146 genes [annotated by UniProt Consortium (2018)], 62% of which significantly influenced patient survival, and a greater proportion of the most highly ranked genes were significantly associated with breast-cancer survival when prioritized with WINNER than with other gene prioritization techniques (PageRank and dual node-edge ranking; Figure 4). Furthermore, the precision of WINNER for retrieving survival-related genes (i.e., the proportion of retrieved genes that were significantly related to breast cancer survival) was even greater when restricted to genes with a ranking *p*-value of *p*_*r*_ < 0.05.

#### WINNER ranking of directed networks

**Figure 5:**
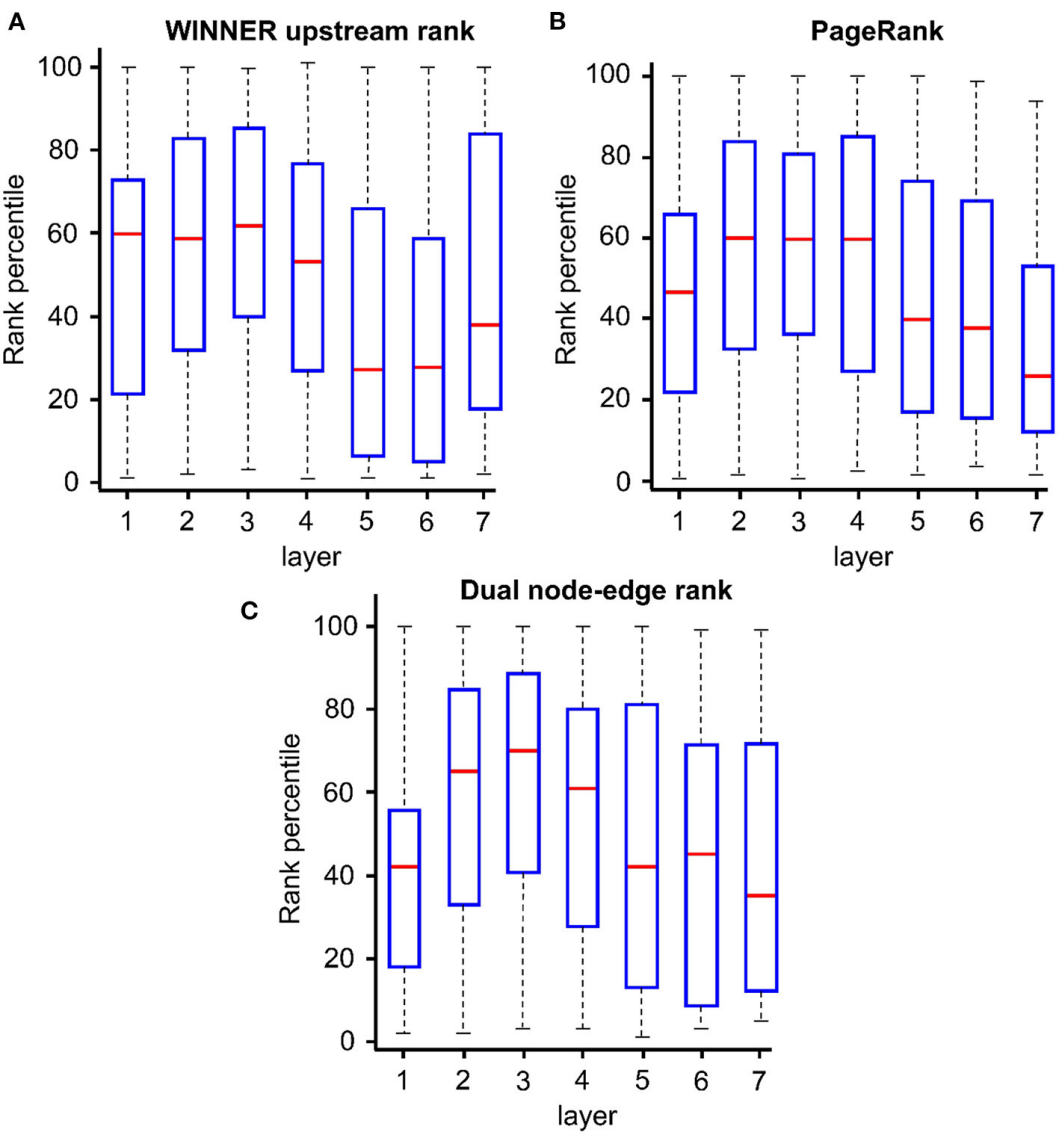
WINNER upstream prioritization more accurately identifies the relative position of genes in a pathway. Gene-gene regulatory relationships from STRING v.10.5 were used to distribute genes from all KEGG cancer pathways into 7 layers *via* WINNER (customized for upstream ranking), PageRank, and Dual Rank; genes coding for proteins that function further upstream in the pathways were assigned to the lower-numbered layers. Layers 1–3 are the most upstream layers, usually correspond to the kineases, grow factors, and receptors. Layers 4–7 are downstream, usually correspond to signaling hubs, phospholization, transcription factors, and inside-nucleus genes. The y axis indicates the ranking scores, which were converted into percentile so that the rankings across different pathways could be combined into one boxplot. The red cross implies boxplot outliners (beyond 2 and 98% percentiles). **(A)** WINNER upstream rank. **(B)** PageRank. **(C)** Dual node-edge rank.

WINNER ranking of directed networks was evaluated *via* WINNER upstream prioritization with all cancer disease pathways in KEGG release 85 (Kanehisa et al., 2017; KEGG, 2022) and the gene-gene regulatory relationships in STRING v.10.5 (Szklarczyk et al., 2017). Genes were distributed into layers using the breadth-first search approach (Wang et al., 2012) with genes coding for proteins that function further upstream in the pathways assigned to the lower-numbered layers. Thus, genes in the lowest-numbered layers tend to encode master regulatory molecules/receptors and first/second messengers, which are located where the signaling cascade originates (e.g., near the cell membrane; Koschmann et al., 2015), while genes with the highest layer numbers tend to encode downstream effector molecules that are closely associated with a specific disease phenotype, such as drug resistance in breast cancer (Johnston, 2006). Our results indicated that using WINNER, layer 1–3 genes, which were the upstream layers in the pathways, were consistently ranked at higher percentiles than genes at other layers (more downstream; Figure 5). But this consistency was not observed when the genes were prioritized *via* equivalent (directed-network ranking) analyses with PageRank (Winter et al., 2012) and dual node-edge ranking (Wang et al., 2015). WINNER upstream overestimated the ranking of genes in layer 8, but this can likely be attributed to noise, because the layer contained only 12 ranked genes.

#### WINNER network expansion and ranking upstream regulators

**Figure 6:**
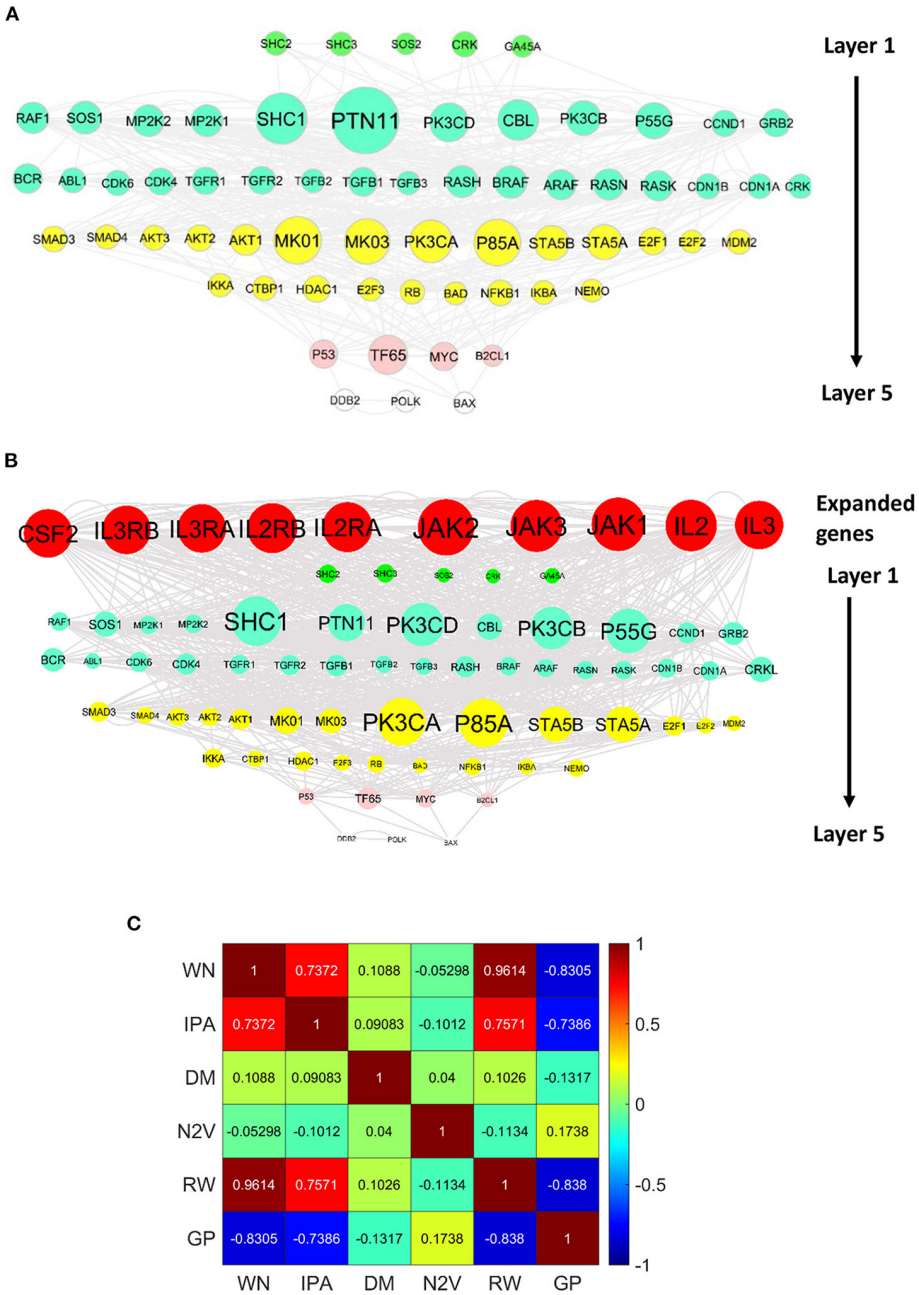
WINNER upstream ranking and expansion can identify genes that are missing from established chronic myeloid leukemia (CML) networks. Genes in the KEGG CML pathways were distributed into layers *via* WINNER upstream, and genes that were missing from the networks were identified *via* WINNER expansion. Genes in the same layer are displayed in the same color, and the size of the node represents the WINNER score. **(A)** WINNER ranking without expansion. **(B)** WINNER ranking with expanded genes. **(C)** Correlation among WINNER (WN), Igenunity Pathway Analysis (IPA), DIAMOnD (DM), Node2Vec (ND), Random Walk (RW), and GenePANDA (GP) ranking.

**Figure 7:**
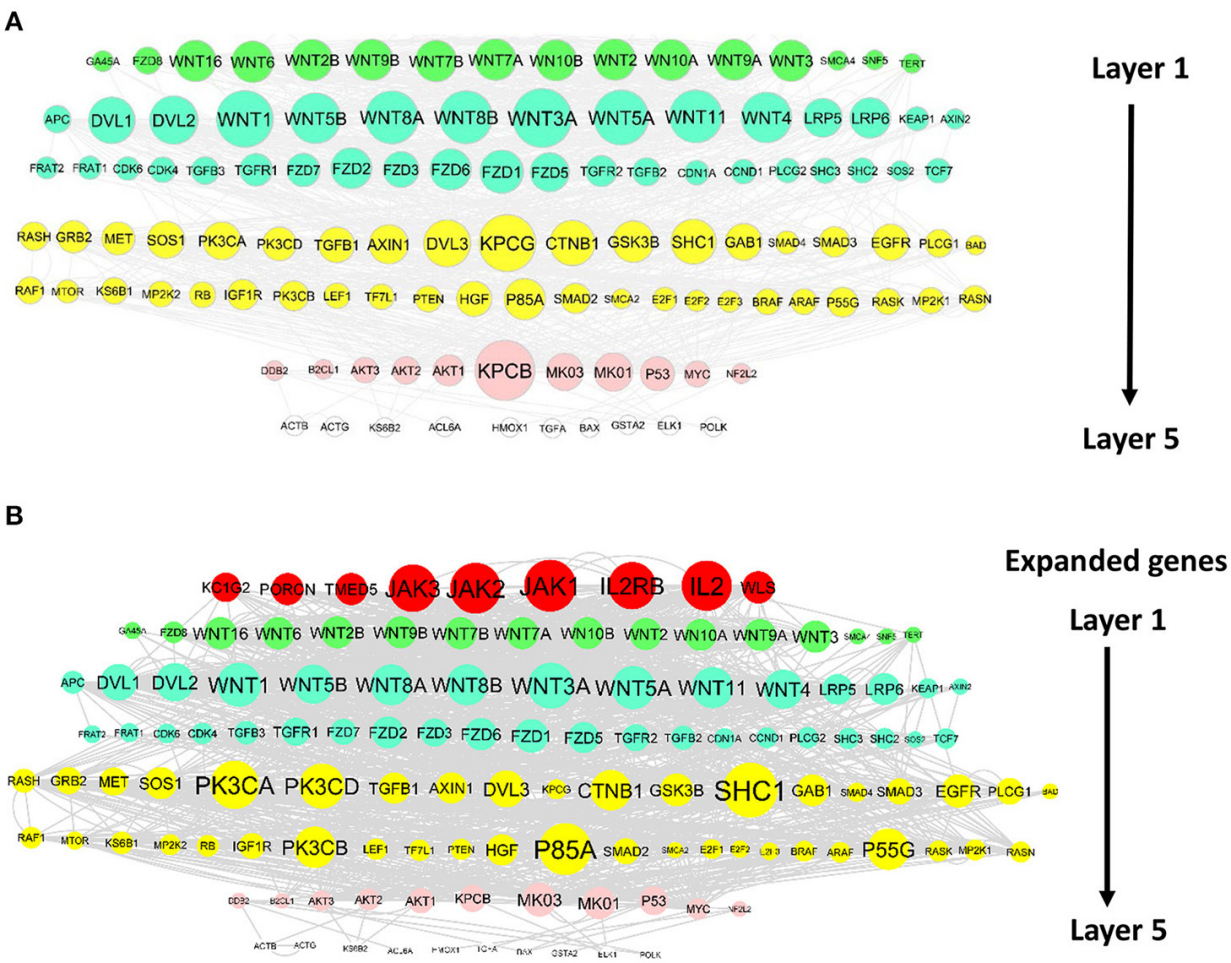
WINNER upstream ranking and expansion can identify genes that are missing from established hepatocellular carcinoma networks. Genes in the KEGG hepatocellular carcinoma pathways were distributed into layers *via* WINNER upstream, and genes that were missing from the networks were identified *via* WINNER expansion. Genes in the same layer are displayed in the same color, and the size of the node represents the WINNER score. **(A)** WINNER ranking without expansion. **(B)** WINNER ranking with expanded genes.

We demonstrated how WINNER could identify upstream regulators of two cancer pathways, Chronic Myeloid Leukemia (CML; https://www.genome.jp/kegg-bin/show_pathway?hsa05220) and hepatocellular carcinoma (https://www.genome.jp/pathway/hsa05225), that were missing from the existing pathways in KEGG but were present in the KEGG database itself. WINNER upstream prioritization distributed genes into five different layers for each pathway, and WINNER expansion added several highly ranked genes to both networks. Additions to the CML network (Figure 6) included JAK1/2/3 and proteins that participate in IL-2 (IL2, IL2RA, and IL2RB), IL-3 (IL-3, IL-3RA, and IL-3RB), and GM-CSF (CSF2) signaling, which is consistent with the JAK2/STAT5 pathway's status as one of the primary targets for treatment of CML (Valent, 2014), as well as evidence that STAT5 is phosphorylated by IL-2 (Kobayashi et al., 2014; Valent, 2014) and IL-3 (Jiang et al., 1999) signaling, and that GM-CSF is a crucial growth factor for myeloid cells; notably, several of these molecules are currently being investigated as therapeutic targets for CML treatment (Hercus et al., 2012; Broughton et al., 2014; Kobayashi et al., 2014). For the hepatocellular carcinoma pathway (Figure 7), WINNER expansion added KC1G2, a serine-threonine kinase that can activate TGF-β1/Smad signaling (Guo et al., 2008); TMED4, WLS, and PRCN, which mediate Wnt/β-catenin signaling (Guo et al., 2008; Martin-Orozco et al., 2019; Bland et al., 2021); and several genes for proteins in the FGF signaling pathway (FRS2, FRS3, KLB, and PLCG1; Gotoh, 2008; Gyanchandani et al., 2013; Wang et al., 2020), of which KLB is particularly important, because it functions as a co-receptor for the binding of FGF-19/21 to FGFR-1/4 (Yang et al., 2012). Thus, the genes added to the KEGG CML and hepatocellular carcinoma pathways by WINNER expansion have strong, well-established links to multiple binding partners that participate in the mechanisms associated these diseases.

Besides, WINNER ranking correlation with other ranking techniques, including Ingenuity Pathway Analysis (IPA; Kramer et al., 2014), DIAMOnD (Ghiassian et al., 2015), Random Walk (Smedley et al., 2014), Node2Vec (Grover and Leskovec, 2016; Peng et al., 2019), and GenePANDA (Yin et al., 2017), vary from −0.83 (negatively correlated) to −0.05 (insignificant correlation), then to 0.74 (moderate-positively correlated; Figure 6C). This result suggests that the major difference between WINNER and other techniques' ranking appears when the network expands beyond the seed genes. Thus, a good benchmark among WINNER and other techniques can be performed by a network-expansion scenario.

### Benchmarking WINNER ranking by retrieving newly updated genes in KEGG pathways

Gene prioritization algorithms are benchmarked by information retrieval experiments, such as in Guala and Sonnhammer (2017) and Zhang et al. (2021), where some important regulators are labeled “unknown,” and the algorithms are executed to rank these “unknown-labeled” gene such that these regulators are top-ranked. Thus, to benchmark WINNER, we setup the KEGG Pathway retrieval experiment. Here, WINNER took a KEGG pathway release 50 (2009 version; Kanehisa et al., 2010) as the seed genes and gene-gene interactions (expanded network) in HAPPI database (Chen et al., 2017) as the input; the WINNER expansion *p*-value (*p*_*e*_) and WINNER score were calculated for candidate genes to include in the KEGG release 50 pathway networks; then, the highly-ranked non-seed (expanded genes) was compared to the same updated pathway network in KEGG release 85 (Ogata et al., 1999; Kanehisa et al., 2017; 2017 version) as the ground-truth. In this experiment, WINNER performance, quantified by precision, recall, and the F1 score, was compared with Ingenuity Pathway Analysis (IPA; Kramer et al., 2014), DIAMOnD (Ghiassian et al., 2015), Random Walk (Smedley et al., 2014), Node2Vec (Grover and Leskovec, 2016; Peng et al., 2019), and GenePANDA (Yin et al., 2017); these techniques were chosen according to Zhang et al. (2021). The same experiment was executed with each KEGG pathway, and the results were aggregated into error bars.

**Figure 8:**
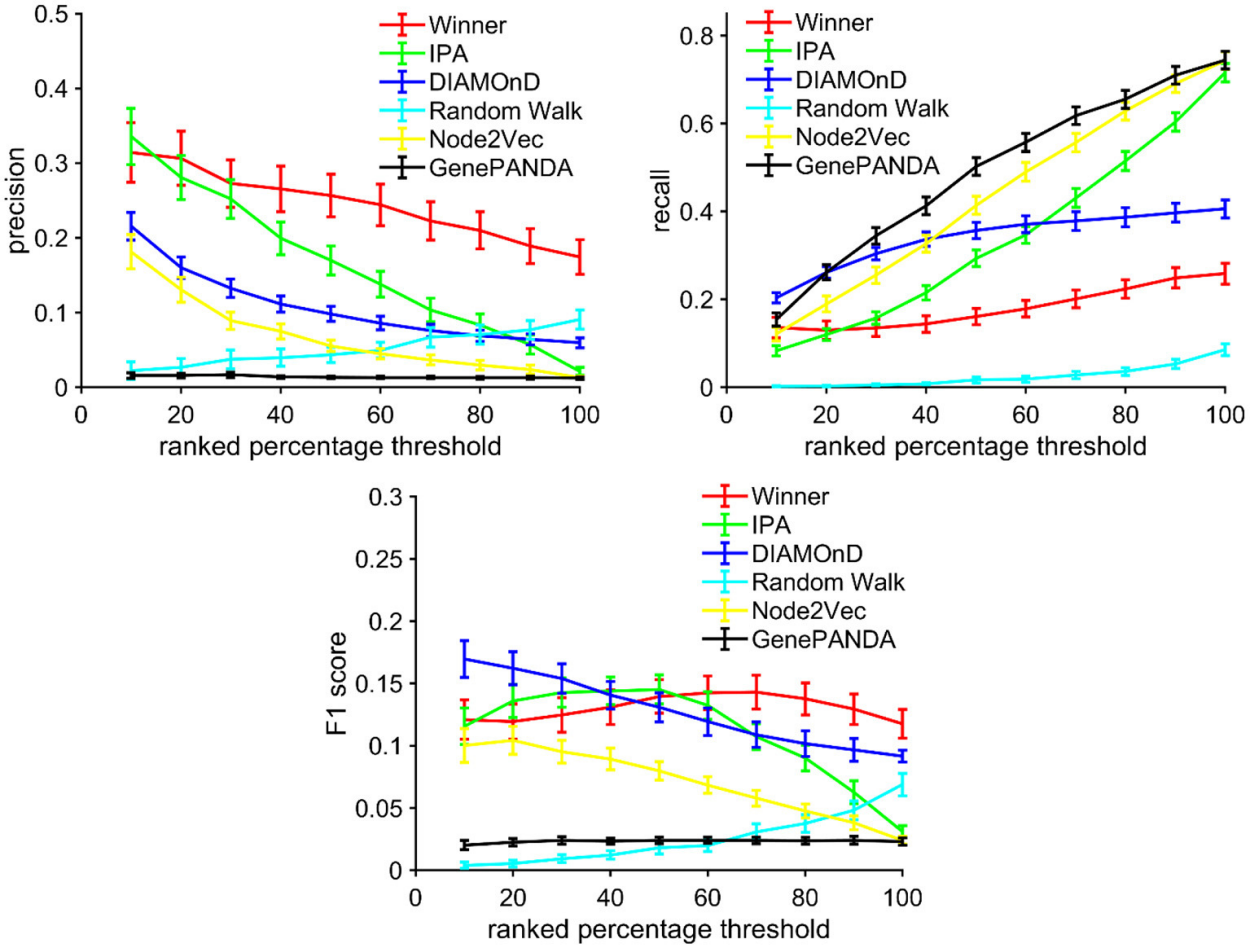
Benchmark: WINNER expansion more accurately identifies the addition of new genes to established networks. The pathway networks in KEGG (https://www.genome.jp/kegg/network.html) release 50 was expanded *via* WINNER (i.e., calculation of the WINNER expansion *p*-value), Ingenuity Pathway Analysis (IPA), DIAMoND, Random Walk, Node2Vec, and GenePANDA. Then, the expanded networks were compared to the updated network in KEGG release 85 to determine the precision, recall, and F1 scores for each expansion technique.

Our results indicated that the WINNER predictions had greater precision but less recall (i.e., the proportion of newly incorporated genes that were retrieved by the prediction) than the predictions generated *via* other comparing methods (Figure 8). The WINNER predictions were also associated with a higher F1 score, which incorporates both precision and recall into a global measure of accuracy, when more than 60% of the extension candidates were examined. Besides, Figure 8 shows that the retrieval recall rate is low (usually < 0.2) in all of the algorithms. Precision should be prioritized in comparing the performance among these expansion algorithms.

### WINNER ranking of differentially expressed genes in biological case-studies

#### WINNER ranking of genes involved in apoptosis and cell-cycle activity

The use of WINNER for prioritizing genes involved in cellular processes was evaluated with the KEGG apoptosis and cell-cycle pathways and node-degree–preserved network randomization. WINNER ranking *p*-values were highly significant for genes that participate in some of the most essential mechanisms of apoptosis, such as Phosphatidylinositol 4,5-bisphosphate 3-kinase catalytic subunit alpha isoform (PIK3CA) (*p*_*r*_ = 5.01 × 10^−13^); the Phosphatidylinositol 3-kinase regulatory subunit alpha (P85A; *p*_*r*_ = 1.34 × 10^−12^) and Cytokine receptor common subunit beta (IL3RB; *p*_*r*_ = 4.60 × 10^−12^); and genes for several proteins of the cytoskeleton (actin, *p*_*r*_ = 1.94 × 10^−104^; Tubulin, *p*_*r*_ = 1.94 × 10^−104^; B4DZT3, *p*_*r*_ = 8.71 × 10^−87^; Lamin A/C, *p*_*r*_ = 8.17 × 10^−87^; Lamin B1, *p*_*r*_ = 8.17 × 10^−87^; actin-G, *p*_*r*_ = 5.15 × 10^−63^), which is substantially reorganized to produce the characteristic shrunken morphology of apoptotic cells; notably, actin and actin-binding proteins also initiate and regulate apoptosis (Desouza et al., 2012). However, the KEGG apoptosis pathway also includes genes for a number of proteins that participate IL-3– and NGF-signaling (IL-3, IL-3R, and NGF), which are nonessential (or even irrelevant) for apoptosis, and the ranking *p*-values calculated for these genes were not significant (*p*_*r*_ = 0.18). Similarly, genes in the KEGG cell-cycle pathway that encode proteins directly involved in DNA replication and cell division had highly significant ranking *p*-values (Cell Division Cycle 14B, *p*_*r*_ = 9.5 × 10^−297^ and 14A, *p*_*r*_ = 2.28 × 10^−22^) whereas the ranking *p*-values for genes that participate in TGF-β signaling were nonsignificant (TGF-β, *p*_*r*_ = 0.29; SMAD2, *p*_*r*_ = 0.29; SMAD3, *p*_*r*_ = 0.29; SMAD4, *p*_*r*_ = 0.29), which is consistent with the role of TGF-β in cell-proliferation: it interacts with many components of the cell cycle pathway but generally inhibits proliferation in non-mesenchymal cells. Collectively, these observations demonstrate that the WINNER ranking *p*-value can be a useful guide for distinguishing between genes that are essential or nonessential participants in a particular cellular process.

#### WINNER ranks important signaling pathway markers in mammalian pig heart regeneration

**Figure 9:**
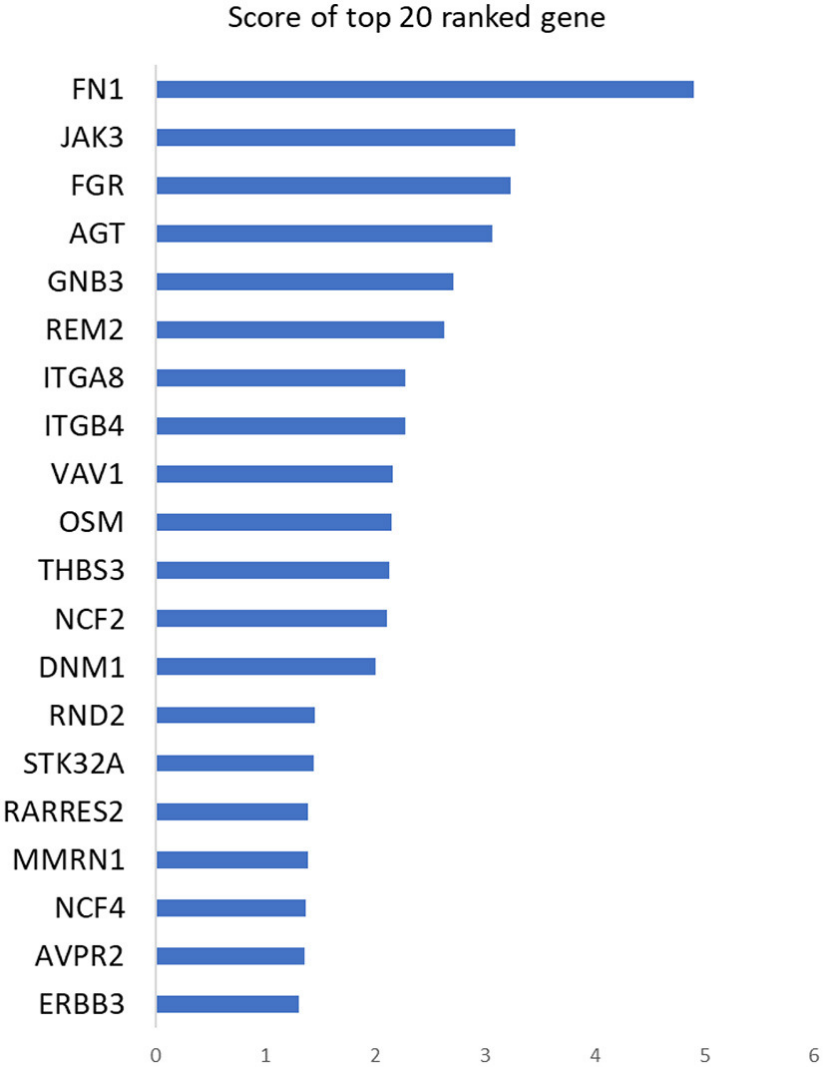
WINNER can identify genes that contribute to cardiac regeneration from a list of differentially expressed genes. RNA-sequencing analyses of gene expression in the hearts of piglets that had or had not undergone surgically induced myocardial infarction on the 1st day after birth for a previous report (Zhu et al., 2018) were compared to generate a list of differentially expressed genes; then their gene-gene interactions were queried from HAPPI v2 database; then, the list was ranked *via* WINNER gene prioritization to determine which genes likely contributed to myocardial regeneration. The 20 top-ranked genes are displayed with their corresponding WINNER scores.

The hearts of adult mammals cannot regenerate myocardial tissues that are lost to injury; however, when myocardial infarction (MI) was induced in the hearts of one-day-old piglets, the animals recovered with no significant loss of cardiac function and little evidence of myocardial scarring (Zhu et al., 2018). Thus, to identify genes that may contribute to mammalian cardiac regeneration, we used WINNER to rank the list of differentially expressed genes from piglets that had or had not undergone surgically induced MI on postnatal day 1 for a previous report (Zhang et al., 2020; Figure 9, Supplementary Table 1). Here, we used HAPPI version 2 database (Chen et al., 2017) to build the network connecting these genes. The two top-ranked genes (FN1 and JAK3) encoded fibronectin, which is required for cardiac regeneration in zebrafish (Wang et al., 2013), and Janus kinase 3 (JAK3), which has been shown to protect against ischemia-reperfusion injury (Kubin et al., 2011); notably, JAK3 also interacts with oncostatin-M, which is encoded by the tenth-highest WINNER-ranked gene (OSM) and is a primary factor in cardiomyocyte dedifferentiation and remodeling (Singh et al., 2016; Doll et al., 2017). Also among the top 10 were genes encoding subunits of the essential matrix proteins integrin alpha (ITGA8) and beta (ITGB4), which are differentially expressed in adult and fetal cardiac fibroblasts and involved in chamber specification of zebrafish hearts (Singh et al., 2016; Doll et al., 2017), while the 11th-ranked gene, THBS3, encodes another extracellular matrix protein, thrombospontin 3, which is a critical [and clinically relevant (Mustonen et al., 2013)] regulator of cell-cell and cell-matrix signaling that appears to impede integrin function and contribute to injury-induced cardiomyopathy in mice (Costa et al., 2014; Porrello and Olson, 2014; Puente et al., 2014). Other genes ranked among the top 20 by WINNER included the nitrous-oxide–related genes NCF2 and NCF4, and the gene for vasopressin 2 (AVPR2), which collectively modulate the cellular environment to promote cardiac regeneration (Costa et al., 2014; Porrello and Olson, 2014; Puente et al., 2014); ERBB3, which encodes a tyrosine kinase that appears to be crucial for embryonic development (Erickson et al., 1997); and genes for a dynamin protein (DNM1) and a Rho GTPase (RND2), which suggests that at least some of the mechanisms of mammalian myocardial regeneration are mediated by vesicle-based signaling.

#### WINNER ranking reflects the important genes supported by co-citations and reveals the upstream events in the r-type pathway-to-pathway network in triple negative breast cancer (TNBC) study

**Figure 10:**
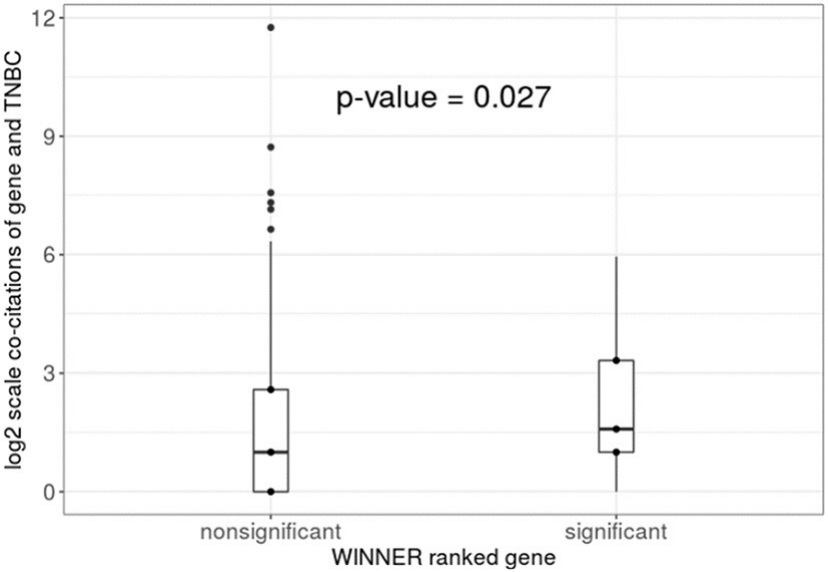
The literature validation of triple negative breast cancer genes using co-citations from PubMed. The co-citations of gene and TNBC are grouped by the WINNER reported *p*-values. The non-significant gene *p*-values are larger than 0.05 in WINNER, and the significant gene *p*-values are ≤ 0.05 in WINNER. The Kruskal Wallis test *p*-value is 0.027.

We found 72 significant genes ranked by WINNER using *p*-value ≤ 0.05 with the WINNER score ranging from 7.4 to 92.5, and the left nonsignificant genes' WINER score ranges from 0 to 68.7. The co-citations analysis shows that the “triple negative breast cancer” co-citations between the significant ranked genes and the nonsignificant ranked genes have significant difference with Kruskal Wallis test's *p*-value = 0.027 (Figure 10). The result suggests that WINNER's high-rank genes are more likely lead to biological insights than the WINNER's low-rank genes.

**Figure 11:**
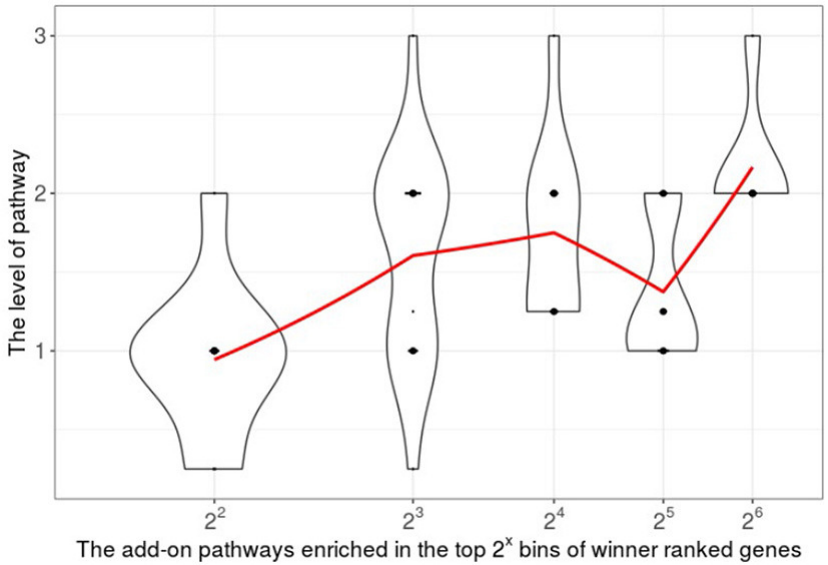
The correlation between the add-on pathways enriched in the top 2^*x*^ bins and the bin size. The violin plot shows the pathway level distribution. The red points connected by solid red lines are the means of pathway levels.

To explore new insights among the high-ranking genes, we performed pathway analysis and built the pathway-to-pathway regulatory networks from these genes using PAGER tool (Yue et al., 2018). The WINNER significantly ranked genes regulated many implicated pathways and processes for TNBC. Thus, we observed the higher ranked gene enriched pathways are more likely to be at upstream side of the regulatory (r-type) enriched pathway-to-pathway network. In general, the add-on pathway levels were positive correlated to the ranked gene bins with Pearson correlation coefficient equal to 0.74 (Figure 11).

We found that the top ranked genes, TOP2A, CDK1, PLK1, and UBE2C, were enriched in the cell cycle related pathways, such as “Phosphorylation of Cyclin B1 in the CRS domain,” “Regulation of mitotic cell cycle,” “Mitotic Metaphase and Anaphase,” and “Free APC/C phosphorylated by Plk1.”

Topoisomerase II a (TOP2A) can be a useful gene in determining whether TNBC patients would have a good response to anthracycline therapy, which is the mainstay treatment in TNBC cancer (Brase et al., 2010; Di Leo et al., 2011; Eltohamy et al., 2018). Both Eltohamy et al. and Di Leo et al. found that patients with aberrant expression of TOP2A have better response to anthracycline treatment (Di Leo et al., 2011; Eltohamy et al., 2018).

Cyclin dependent kinase 1 (CDK1) play a critical role how the cell cycle is regulated, specifically during mitosis. Liu et al. used nanoparticles with siRNA to target CDK1, and it has been found to successfully inhibit the TNBC cell line that has been injected in mice (Liu et al., 2014). Xia et al. has found that the CDK1 inhibitor can inhibit the growth of the TNBC cells by arresting them in the G2/M cell phase (Xia et al., 2014).

Polo like kinase-1 (PLK1) has been found to be one of the key regulators in the cell cycle. Targeting and knocking out of PLK1 has been found to cause the TNBC tumor cells to be arrested in the G2-M cell cycle (Ueda et al., 2019; Zhao et al., 2021; Patel et al., 2022). Morray et al. found that a nanoparticle with siRNA targeting PLK1 can inhibit growth in the TNBC tumor cell line (Morry et al., 2017). Patel et al. used the allosteric inhibitor RK-10 to target the PLK1 in TNBC cell lines, and it has inhibited growth through the S phase and G2/M (Patel et al., 2022).

Overexpression of Ubiquitin-conjugated enzyme (UBE2C) can play a role in the pathogenesis of TNBC (Chou et al., 2014; Kim et al., 2019). Chou et al had found that UBE2C has been highly expressed in cancer tissue cells, and that when UBE2C has been targeted with siRNA, the tumor cells have stopped proliferating (Chou et al., 2014).

## Discussion and conclusion

In this paper, we introduce WINNER, a new network-based ranking tool that addresses several of the limitations associated with other gene prioritization techniques. Our novel use of node-degree–preserved and modularity-preserved randomization produced randomized networks that retained some of the original network topology and were more normally distributed, which increased the precision and robustness of our ranking *p*-value (*p*_*r*_) calculations, while the expansion *p*-value (*p*_*e*_) better accommodated the incomprehensiveness and redundancy of the input gene list. However, WINNER rankings were not well-correlated with the clustering coefficient, which represents the presence of network cliques (Newman, 2008; i.e., semi-isolated groups of genes that collectively function like a single node), which suggests that WINNER ranking may be somewhat compromised in dense networks, such as those containing families of proteins, where the scale-free property (Timar et al., 2016) does not apply. Nevertheless, many biological networks are scale-free (Khanin and Wit, 2006), and since degree-preserved randomization tends to produce near-normal ranking distributions, the WINNER *p*_*r*_ value is likely more accurate than the empirical *p*-value, even for networks that are not perfectly scale-free.

WINNER network ranking belongs to the “eigenvector ranking” (Newman, 2008) class of algorithm. Therefore, it has the same “big-O” computational cost to PageRank [O(N^3^), where N is the number of network genes] if implemented using iterative matrix multiplication. However, this class of algorithm can be implemented in parallel, which significantly reduced the computational time in practice.

The performance of gene network prioritization significantly depends on the disease (Zhang et al., 2021), or the biological case-study. Therefore, we demonstrate WINNER's performance in various disease and biological study scenarios. The comprehensive KEGG pathway results reflect the case when lacking biological samples and expression data. Then, prioritization needs to be performed only using the domain-knowledge available network to generate hypotheses. Cardiac regeneration, which focuses on cardiomyocyte proliferation, case-study is an example when a significant biological process, not a disease, that does not naturally happen in matured mammals (Porrello et al., 2011; Lam and Sadek, 2018; Ye et al., 2018; Zhu et al., 2018; Zhao et al., 2020; Nakada et al., 2021; Nguyen et al., 2022). In this case, the focus is finding the regulating mechanism to create new cells and to apply this knowledge in biomedical engineering research. Cancer and other disease case studies (leukemia, TNBC, and Vitamin D) are directly related to the disease, and targeted therapies to kill cells are available or proposed. In this case, the focus is to find markers, especially the “cell-killer ones” associated with the disease outcomes, and there is less emphasis rather than the regulating growing mechanism. WINNER results are insightful in all of these cases, whereas whether other techniques have insightful results is yet to be examined in multiple studies.

In conclusion, WINNER gene prioritization is generally more accurate and robust than other network-based prioritization techniques, such as PageRank and node-degree ranking, and can be effective for identifying genes that may be missing from established gene networks, for determining the relative position (i.e., upstream or downstream) of genes within a pathway, and for ranking a list of differentially expressed genes. The superior performance is linked to better retrieval precision when expanding the network among the seed genes. The important case studies presented in this work are in a scenario where new disease-specific gene-expression data were generated, and novel genes associated with the disease and phenotype are expected. Then, network expansion is required. In this expansion, WINNER emphasizes precision, where only a small expanded but highly relevant candidates are explored, over recall, where more comprehensive candidate genes were explored but may involve many irrelevant ones. Other methods tend to emphasize recall; therefore, they may computationally retrieve more candidates; however, at the same time, make it much more difficult for the user to choose the rightly relevant ones. Also, having too many irrelevant genes in the network significantly affects the ranks of the well-known disease-specific genes. This scenario explains the advantage of WINNER over other methods. Future investigations are warranted to determine what additional biological insights can be obtained by using WINNER to rank genes that participate in other cellular processes, in metabolic regulatory pathways (Berkhout et al., 2013), and in co-expression networks (Radulescu et al., 2018).

## Data availability statement

The gene expression data used in this work are publicly available at the Gene Expression Omnibus database, accession number GSE144883, https://www.ncbi.nlm.nih.gov/geo/query/acc.cgi?acc=GSE144883.

## Author contributions

TN developed the algorithm, performed case studies, and wrote the manuscript. ZY and RS performed case studies and performed the literature validation of the results. ZY built the website. RW and JZ provided data and participated in the case studies. JC conceptualized the ideas, helped design the analytical experiments, and revised the final manuscript. All authors read, edited, and approved the manuscript.

## Funding

The work was in part supported by the internal University of Alabama at Birmingham research grants to JC, the National Institutes of Health grant awards U54TR001005 in which JC serves as a co-investigator, and R01 awards R01HL150078 in which RW serves as principle investigator and JC serves as co-investigator.

## Conflict of interest

The authors declare that the research was conducted in the absence of any commercial or financial relationships that could be construed as a potential conflict of interest.

## Publisher's note

All claims expressed in this article are solely those of the authors and do not necessarily represent those of their affiliated organizations, or those of the publisher, the editors and the reviewers. Any product that may be evaluated in this article, or claim that may be made by its manufacturer, is not guaranteed or endorsed by the publisher.

## Footnotes

^1^chi2gof: Chi-square goodness-of-fit test [https://www.mathworks.com/help/stats/chi2gof.html].

## Supplementary material

Supplementary Figure 1 Schematic diagrams of WINNER gene prioritization and network expansion. **(a)** Seeded genes (green) and candidate expansion genes (yellow) are assembled into a network as indicated by their pairwise interactions. **(b)** The expansion *p*-value (*p*_*e*_) are calculated among the expansion-candidate genes, then genes with *p*_*e*_ < 0.05 will be further evaluate and added into and expand the network, one gene at a time. Then **(c)** the expansion score **(e)** are calculated for the candidate expansion genes; then, the highest-scored gene is added to the network; this process is repeated until all candidates are added or being halted (not adding all candidates). And **(d)**, after completing the expansion, the statistical significance of the rankings are recalculated for the expanded network.

Supplementary Figure 2 WINNER filtering of candidate genes for network expansion. Red nodes represent seeded genes, open nodes represent candidate expansion genes, black lines represent interactions between two seeded genes, and gray lines represent interactions between one seeded gene and one expansion gene or between two expansion genes. Candidate genes for network expansion were filtered *via* two tests: (1) the likelihood of the candidate expansion gene (E.Gene) having a seeded interaction relative to its total number of interactions (bottom left table), and (2) the likelihood of the candidate expansion gene having seeded interactions relative to the seeded interactions of its most similar seeded gene (S.Gene), with similarity determined by node degree (bottom right table).

Supplementary Figure 3 WINNER ranking of the network of Alzheimer's disease pathways in KEGG release 50. The network graph was constructed with Cytoscape (Shannon et al., 2003) version 3.6.0 and the force-directed layout; the size of the node represents the WINNER score.

Supplementary Table 1 WINNER ranking for genes in cardiac regeneration dataset. The table includes gene symbol, the indication of whether a gene is a seeded (S) or expanded (E) gene, and WINNER score.

Supplementary Table 2 Gene-gene interaction network in the cardiac regeneration dataset.

Supplementary Table 3 WINNER ranking for genes in triple negative breast cancer (TNBC) dataset. The table includes gene symbol, the indication of whether a gene is a seeded (S) or expanded (E) gene, WINNER score, and *p*-value.

Supplementary Table 4 Gene-gene interaction network in triple negative breast cancer (TNBC) dataset.

Supplementary Video 1 The .cys (cytoscape) file of the regulatory (r-type) pathway-to-pathway network in the triple negative breast cancer study.
