# Supplementary material for: WINNER: A network biology tool for biomolecular characterization and prioritization
Source: Front Big Data. 2022 Nov 4;5:1016606. doi: 10.3389/fdata.2022.1016606 (PMC9672476; doi:10.3389/fdata.2022.1016606)
Supplement: Supplementary Figure 1 — Schematic diagrams of WINNER gene prioritization and network expansion. (a) Seeded genes (green) and candidate expansion genes (yellow) are assembled into a network as indicated by their pairwise interactions. (b) The expansion p-value (pe) are calculated among the expansion-candidate genes, then genes with pe < 0.05 will be further evaluate and added into and expand the network, one gene at a time. Then (c) the expansion score (e) are calculated for the candidate expansion genes; then, the highest-scored gene is added to the network; this process is repeated until all candidates are added or being halted (not adding all candidates). And (d), after completing the expansion, the statistical significance of the rankings are recalculated for the expanded network. [file Image_1.pdf]

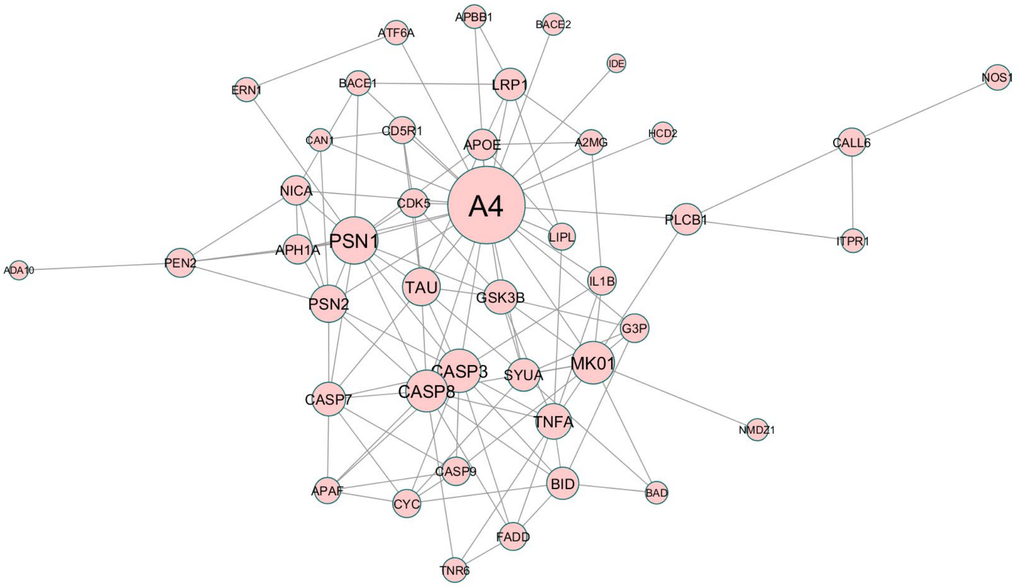

Supplemental Figure 1

Illustration of Winner ranking result for the Alzheimer's disease pathway in KEGG release 50. Here, the node side reflects the Winner score. We visualize the graph using Cytoscape [1] version 3.6.0 and the force-directed layout.
