# Supplementary material for: WINNER: A network biology tool for biomolecular characterization and prioritization
Source: Front Big Data. 2022 Nov 4;5:1016606. doi: 10.3389/fdata.2022.1016606 (PMC9672476; doi:10.3389/fdata.2022.1016606)
Supplement: Supplementary Figure 2 — WINNER filtering of candidate genes for network expansion. Red nodes represent seeded genes, open nodes represent candidate expansion genes, black lines represent interactions between two seeded genes, and gray lines represent interactions between one seeded gene and one expansion gene or between two expansion genes. Candidate genes for network expansion were filtered via two tests: (1) the likelihood of the candidate expansion gene (E.Gene) having a seeded interaction relative to its total number of interactions (bottom left table), and (2) the likelihood of the candidate expansion gene having seeded interactions relative to the seeded interactions of its most similar seeded gene (S.Gene), with similarity determined by node degree (bottom right table). [file Image_2.pdf]

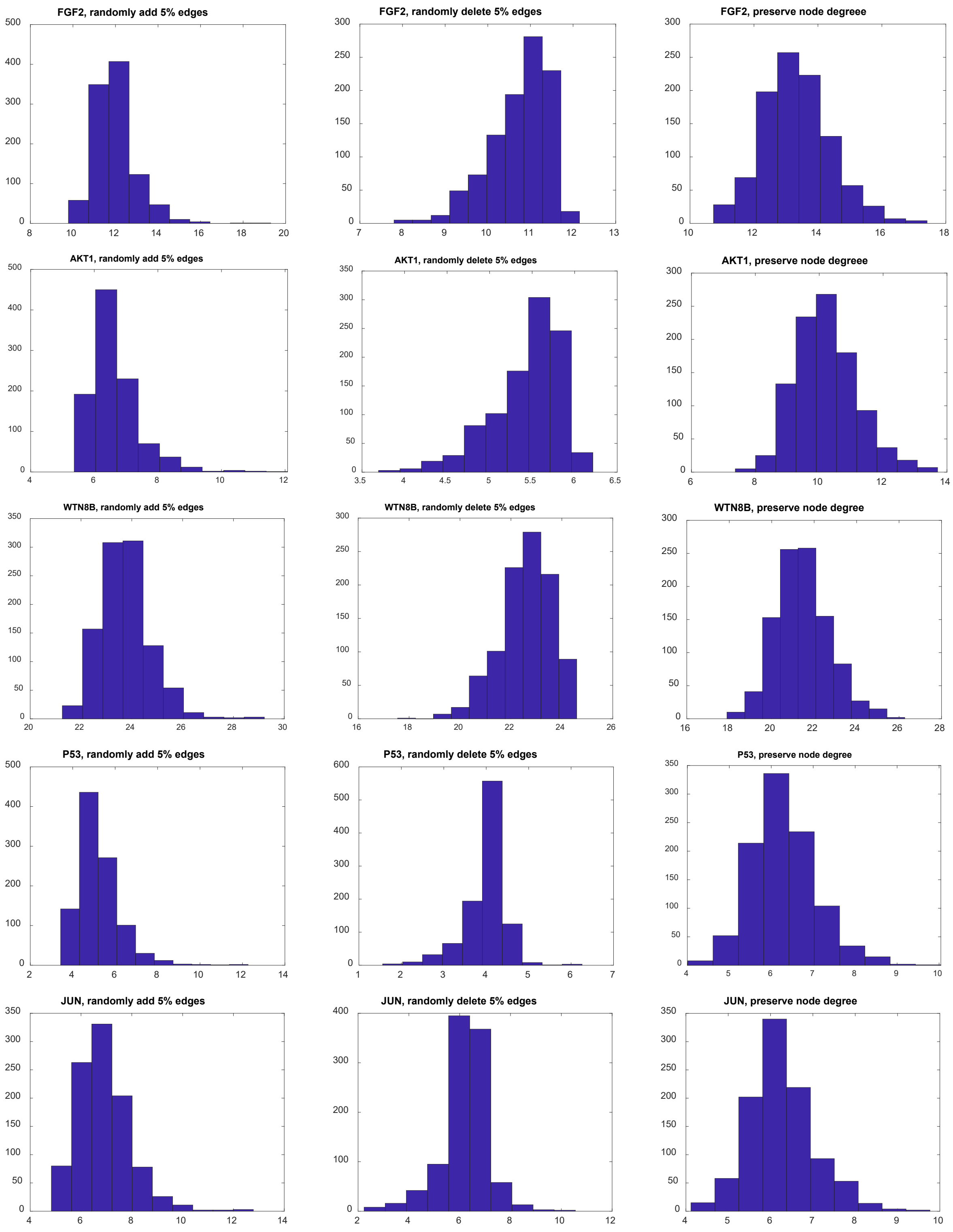

Supplemental Figure 2

Illustration of winner ranking score distribution for different genes (chosen randomly) in ranking Breast Cancer pathway gene (https://www.genome.jp/kegg-bin/show_pathway?hsa05224) using different network randomization approaches. The left/middle/right column is for randomly

adding/removing 5% of the edges and preserving node degree.
