# Supplementary material for: WINNER: A network biology tool for biomolecular characterization and prioritization
Source: Front Big Data. 2022 Nov 4;5:1016606. doi: 10.3389/fdata.2022.1016606 (PMC9672476; doi:10.3389/fdata.2022.1016606)
Supplement: Supplementary Figure 3 — WINNER ranking of the network of Alzheimer's disease pathways in KEGG release 50. The network graph was constructed with Cytoscape (Shannon et al., 2003) version 3.6.0 and the force-directed layout; the size of the node represents the WINNER score. [file Image_3.pdf]

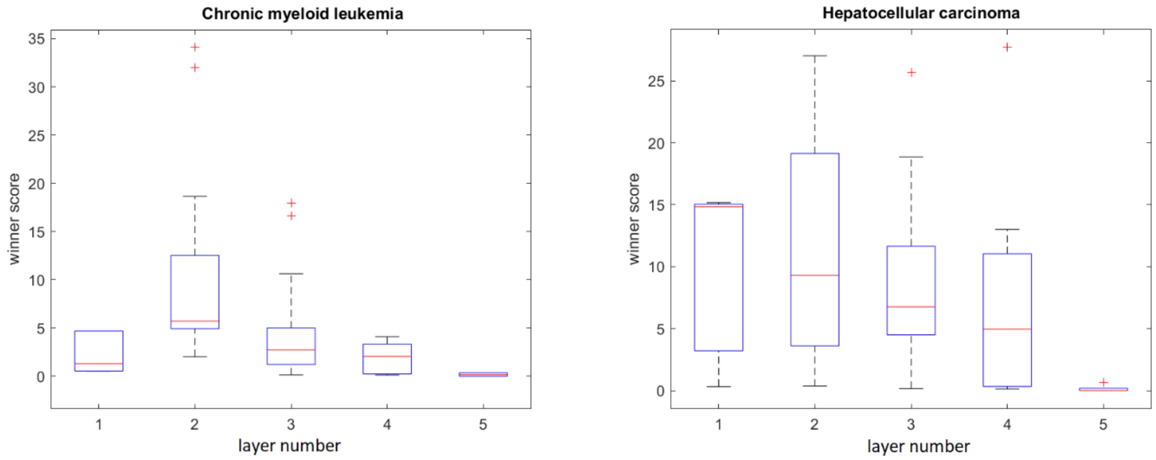

Supplemental Figure 3

Supplemental Distribution of winner score for genes belonging to different layers: Chronic myeloid leukemia (left), Hepatocellular carcinoma (right).
